# Copy number variation (CNV) in the *IGF1R* gene across four cattle breeds and its association with economic traits

**DOI:** 10.5194/aab-62-171-2019

**Published:** 2019-04-11

**Authors:** Yi-Lei Ma, Yi-Fan Wen, Xiu-Kai Cao, Jie Cheng, Yong-Zhen Huang, Yun Ma, Lin-Yong Hu, Chu-Zhao Lei, Xing-Lei Qi, Hui Cao, Hong Chen

**Affiliations:** 1 College of Animal Science and Technology, Northwest A&F University, Yangling Shaanxi, 712100, P. R. China; 2 College of Life Sciences, Xinyang Normal University, Institute for Conservation and Utilization of Agro-Bioresources in Dabie Mountains, Xinyang, Henan, 464000, P. R. China; 3 Key Laboratory of Adaptation and Evolution of Plateau Biota, Northwest Institute of Plateau Biology, Chinese Academy of Sciences, Xining, Qinghai, 810001, P. R. China; 4 Bureau of Animal Husbandry of Biyang County, Biyang, Henan, 463700, P. R. China; 5 Shaanxi Kingbull Animal Husbandry Co. Ltd., Yangling, Shaanxi, 712100, P. R. China

## Abstract

The insulin-like growth factor 1 receptor (IGF1R) plays a vital role in immunomodulation and muscle and bone growth. The copy number variation (CNV) is believed to the reason for many complex phenotypic variations. In this paper, we statistically analyzed the copy number and the expression profiling in different tissue types of the *IGF1R* gene using the 422 samples from four Chinese beef cattle breeds, and the mRNA of *IGF1R* was widely expressed in nine tissue types of adult cattle (heart, liver, kidney, muscle, fat, stomach, spleen, lung and testis). Results of CNV and growth traits indicated that the *IGF1R* CNV was significantly associated with body weight and body height of Jinnan (JN) cattle and was significantly associated with body height and hucklebone width of Qinchuan (QC) cattle, making *IGF1R* CNV a promising molecular marker to improve meat production in beef cattle breeding. Bioinformatics predictions show that the CNV region is highly similar to the human genome, and there are a large number of transcription factors, DNase I hypersensitive sites, and high levels of histone acetylation, suggesting that this region may play a role in transcriptional regulation, providing directions for further study of the role of bovine CNV and economic traits.

## 1 Introduction

With the development of industry and the improvement of living standard, beef has gradually made up a greater proportion of the food consumed (Barendse et al., 1997). The economic performance of production traits can be improved by continuous breeding. Various genes regulate meat quality traits, so we can study their genetic variation for marker-assisted selection (MAS) (Cooper et al., 2011). MAS is a breeding method which genotypes molecular markers in larger populations, and these markers are usually linked to causal mutations (Ruane et al., 2007). For example, single nucleotide polymorphism (SNP) and insertion/deletion (Indel) have been used to explore growth traits of cattle in genomic regions (Jin et al., 2016; Pan et al., 2013). However, the above methods also have certain limitations. The effect of many SNPs and Indels, presenting one point or a small mutation, have proved to be insufficient as a cause of phenotype alteration (Huaixing et al., 2008; Sun et al., 2012). So, to speed up molecular breeding, we should choose more effective methods in genomic variations.

Copy number variation (CNV) as a new form of genetic variation is defined as the insertion or deletion of more than 50 bp at the genome level between two individuals of the same species (Mills et al., 2011). In addition, CNV is considered to have influenced many mammalian phenotypes, and it has emerged that the dose effect can affect the expression level of the dose-sensitive genes. This may also be due to other factors such as the fusion of genes, gene function blocking, the location effect, and the removal effect of recessive alleles (Conrad et al., 2010; Kurotaki et al., 2005). CNVs as genomic structural variations contain much more bases, so a stronger genetic impact may arise. In humans, large-scale analyses of CNV data have found that some genes are linked to human diseases, such as *C4* in systemic lupus erythematosus, *FCGR3B* in glomerulonephritis, *hBD-1* in psoriasis, and *CCL3L1* in HIV/AIDS (Fanciulli and Al, 2007; Gonzalez et al., 2005; Hollox et al., 2008; Yang et al., 2007).

The insulin-like growth factor-1 receptor (*IGF1R*), a member of *IGF* gene family, plays essential roles in embryo stages and individual growth after birth (Savage et al., 2010; Ziv and Hu, 2011). IGF1R is the receptor with which IGFs perform biological effects. It can regulate IGF half-life and activity, and it plays a very important role in immune regulation, the formation of lymphocyte, and muscle and bone growth (Adams et al., 2000; Chen et al., 2012). Previous articles have reported that *IGF1R* gene polymorphism could affect the growth traits of different species, providing theoretical support for genetic improvement (Proskura and Szewczuk, 2014; Roldan et al., 2007; Szewczuk et al., 2013). It is worth mentioning that *IGF1R* regulates cell proliferation and apoptosis as a vital target to treat cancer (Baserga et al., 2003; Neuzillet et al., 2017). Meanwhile, a high *IGF1R* gene copy number may have a positive effect on treating cancer (Dziadziuszko et al., 2010).

Thus, we use the *IGF1R* gene as a candidate gene to study the correlation between the gene copy number variation and the growth traits of Chinese cattle, so as to promote the genetic improvement of beef cattle.

## 2 Materials and methods

### 2.1 Samples and trait record

There is currently a commitment to improve the beef quality in Chinese cattle under intensive animal husbandry, and in order to made a thorough inquiry of copy number variation in the bovine *IGF1R* gene, this study used a total of 422 blood or ear samples of female cattle. The cattle sampled were three endemic beef cattle breeds in China (Qinchuan cattle, QC; Jinnan cattle, JN; Nanyang cattle, NY) and a new beef cattle breed (Xianan cattle, XN) derived from selected crossbreeding between French Charolais (male) and NY cattle (female). The 422 head of cattle were in the same feeding environment and were forage-fed by leisurely grazing from weaning at 6 months of adulthood. None of the cattle were genetically related. Then we used nine kinds of adult QC bovine tissue types (n=3), including heart, liver, kidney, stomach, muscle, lung, spleen and fat for expression pattern analysis. In addition, the body sizes of QC (body height (BH), hip height (HH), body slanting length (BSL), chest width (CW), rump length (RL), hucklebone width (HW), and body weight (BW)), JN (body height (BH), hip height (HH), body slanting length (BSL), chest width (CW), rump length (RL), and body weight (BW)) and XN (body height (BH), hip height (HH), body slanting length (BSL), chest width (CW), cannon circumference (CC), and body weight (BW)) were recorded for further association analysis (Gilbert et al., 1993). All experiments were approved by the Northwest A&F University Ethics Committee.

### 2.2 Genomic DNA/total RNA isolation

This study used a standard phenol–chloroform protocol to extract genomic DNA from blood and ear tissue (Welter, 1989). As per the instruction manual, total RNA was isolated from tissue by using a TRIzol reagent and treated with RNase-free DNase (TaKaRa, Dalian, China). The quality of RNA was evaluated via 1 % agarose gel electrophoresis, and nanodrop 2000 was used to measure RNA concentration. In addition, this study used PrimeScrip RT Reagent Kit to obtain cDNA (Clontech, TaKaRa). In the end, the DNA and cDNA were diluted to 25 ng µL${}^{-1}$ and then kept in the -80 ${}^{\circ }$C freezer.

### 2.3 Copy number variation and mRNA profiling

Using genomic quantitative polymerase chain reaction (qPCR), the bovine basic transcription factor 3 (*BTF3*) gene was the housekeeping gene to validate the copy number of *IGF1R*, and the β-*actin* gene was used as a housekeeping gene for the tissue distribution profile. In addition, we used the primer 5.0 software to design the primer named IGF1R-P1 from the CNV region of the *IGF1R* DNA base sequence and to design the primer named IGF1R-P2, which was must span an exon–exon junction from the region of the *IGF1R* mRNA base sequence. The primer *BTF3* and β-*actin* were also designed by same software (Table 1). It was worth noting that the semiquantitative method was used to measure the quality of primers before qPCR by Bio-Rad CFX 96^™^ RealTime Detection System (Bio-Rad, Hercules, CA). A total of 12.5 µL reaction mixtures contained 25 ng of genomic DNA/cDNA, 5 pmol of primers 6.25 µL 2*RealStar Green Power Mixture (Genestar, Beijing, China), and 4.25 µL ${\mathrm{ddH}}_{2}O$. Meanwhile, this system ran at one cycle of 10 min at 95 ${}^{\circ }$C and then 40 cycles of 15 s at 95 ${}^{\circ }$C, and 1 min s at 60 ${}^{\circ }$C. For the melting curve, this was one cycle for 1 min at 95 ${}^{\circ }$C and then 1 min at 55 ${}^{\circ }$C; then, there was an increase at a rate of 0.5 ${}^{\circ }$C per cycle to 95 ${}^{\circ }$C. Finally, this was repeated three times for every sample and the mean value of intensity ratios ±SD was calculated for further statistical analysis. Amplification efficiencies for both the target and the internal reference were > 97 % using the standard curve method with four serial dilution points (pooled cDNA or DNA concentration ranging from 500 to 50 pg).

**Table 1:** Primer pairs designed for the genes used in this study.

	Gene	Primer pair sequences (5${}^{\prime }$–3${}^{\prime }$)	Amplification
			length (bp)
DNA level	IGF1R-P1	1F: GACTATGGCACCAGTGTTTGT	171
		1R: CCTTGAGGCTATCGCTGTATT	
	BTF3	2F: CAAGAAGACTCATTCCTT	109
		2R: CACAAGCACATTATTCAC	
mRNA level	IGF1R-P2	3F: CTCAAGGACGGAGTCTTCACC	106
		3R: CATTGGACAGGCCCTGATAC	
	β-actin	4F: CTTCCTGGGCATGGAATCCTG	103
		4R: CAGCACCGTGTTGGCGTAG	

Note: F – forward primer; R – reverse primer.

### 2.4 Bioinformatics prediction of *IGF1R* CNV

Cellular gene expression was critically determined by DNase I hypersensitive sites and sequence-specific transcription factors (TFs) as well as chromatin modifications. We assumed that these regulatory patterns may be located on the *IGF1R* CNV locus, so we used the sequence of CNV on Cow June 2014 (Bos_taurus_UMD_3.1.1/bosTau8) assembly compared with the whole-genome sequence on humans February 2009 (GRCh37/hg19) assembly to predicted these regulatory patterns.

### 2.5 Statistical analysis

This study used the formula $2\times 2^{-\Delta^{\mathrm{Ct}}}$ to confirm the copy number of *IGF1R*, where ΔCt was the mean of the target gene minus the mean of the housekeeping gene (Bae et al., 2010). In addition, we used the ANOVA method in the SPSS software to clarify the association between the copy number of the *IGF1R* gene and the growth traits among the four breeds. According to the hypothesis that there were two copies of DNA in the control region for autosomes, the quantity of the copy number was divided into three types: loss type< 2; normal type: = 2; gain type: > 2 (Yi et al., 2015). Notably, the effects of farm, sex, and season of birth (spring versus fall) did not have any obvious significance for variability of traits in the four breeds as has been previously reported (Cao et al., 2016; Liu et al., 2014, 2016a; Ma et al., 2011). Finally, the following model was used: $Y_{ijk}=\mu +A_{i}+$ CNV${}_{j}+e_{ijk}$, where $Y_{ijk}$ is the observation of the growth traits, μ is the overall mean of each trait, $A_{i}$ is the effect due to ith age, CNV${}_{j}$ is the fixed effect of jth CNV type of *IGF1R*, and $e_{ijk}$ is the random residual error (Xu et al., 2013).

## 3 Results

### 3.1 Distribution of CNVs of *IGF1R* in four cattle breeds

**Figure 1:**
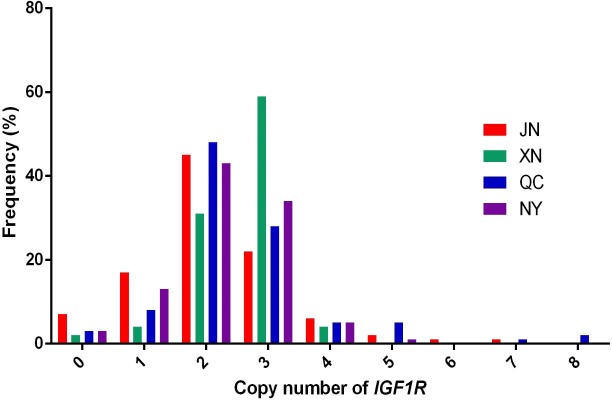
Copy number distributions of *IGF1R* in four Chinese cattle breeds. Histograms show the frequency of individuals with a different copy number of the *IGF1R* gene. Copy numbers were rounded to the nearest integer.

**Figure 2:**
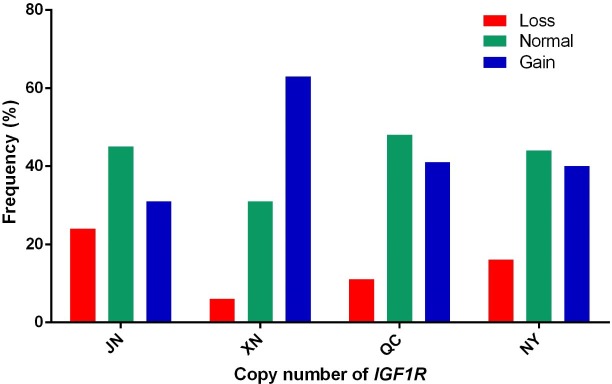
Copy number distributions of *IGF1R* in four Chinese cattle breeds. Histograms show the frequency of individuals with a different copy number type of the *IGF1R* gene. Copy numbers were rounded to the nearest integer.

In our previous work, the whole-genome CNV regions were detected by the custom comparative genomic hybridization (CGH) array (NimbleGen, Roche, Madison, WI, USA) in Chinese cattle (Zhang et al., unpublished results). The analysis found that *IGF1R* covered the CNVR337 region. Specific primers of *IGF1R* were designed based on the NCBI Bos_taurus_UMD_3.1.1 dbVar assembly. To explore the polymorphism of the copy number (CN) in four cattle breeds, we used two ways to show the condition. The CNV types were classified as loss (CN < 2), normal (CN = 2), and gain (CN > 2) according to $2\times 2^{-\Delta^{\mathrm{Ct}}}$. As shown in Fig. 1, the frequencies of copy number polymorphisms in the four cattle breeds had shown which two and three copy numbers take up the large proportion. In addition, as shown in Fig. 2, the three types of *IGF1R* CNV frequency show two results: the normal type was maximal and the gain type was more frequent than the loss type in JN, QC, and NY breeds all the time, but XN cattle were different from the three breeds in that their gain type was maximal.

### 3.2 Gene expression profiling of *IGF1R*

**Figure 3:**
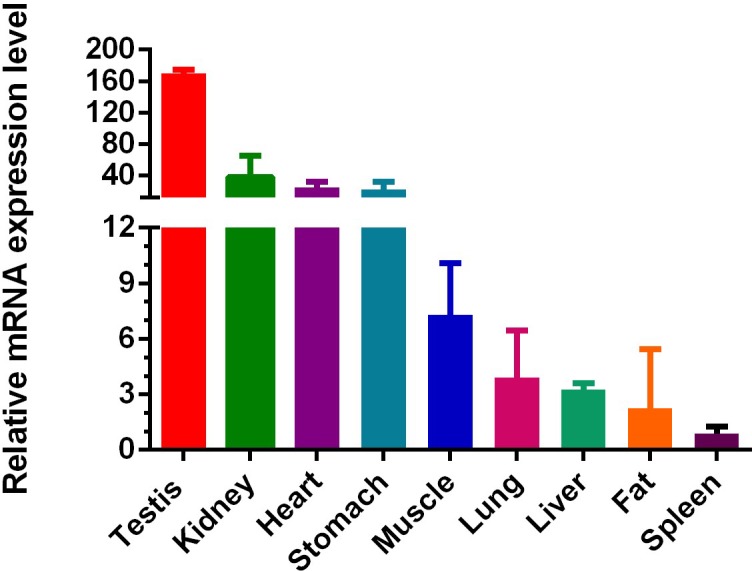
Expression profiling of *IGF1R* in different tissue types of adult cattle. The values are the average of three independent experiments measured by $2^{-\Delta \Delta {}^{\mathrm{Ct}}}$. The relative mRNA expression levels of *IGF1R* are normalized to β-actin. Testicular tissue is from adult male QC cattle, and other tissue is from adult female QC cattle.

*IGF1R* played an important role in immunomodulation, lymphocyte generation, muscle and bone growth. However, there were few reports of the *IGF1R* gene expression in cattle, so we used QC tissue to explore gene expression as shown in Fig. 3. We examined nine types of tissue in total in adult cattle: heart, liver, kidney, muscle, fat, stomach, spleen, and lung (from female cattle) and testis (from male cattle). At the adult stage, the mRNA of *IGF1R* was widely expressed in the nine tissue types, with the highest level of expression in testis. In addition, the level of expression in the kidney, heart, and stomach were second only to the testis, and the middle-level expression was muscle. In the end, low levels of *IGF1R* were measured in liver, fat spleen, and lung.

### 3.3 Correlation analysis of *IGF1R* CNVs and mRNA expression levels

According to the spatiotemporal expression profiling of the *IGF1R* gene, we next analyzed the correlation of *IGF1R* CNVs with mRNA expression levels in testis from 32 adult animals. The reason for using testis was limited by laboratory conditions, and it was highly expressed in male cattle. However, although three types of CNV existed and the mRNA expression varied from 0.17- to 9.43-fold among the tested individuals, the result was found to show no significant correlation between these two data (P=0.079).

### 3.4 Associations between *IGF1R* CNVs and growth traits in all cattle

To explore the relationship between the CNV and growth traits, we used genomic DNA from JN (n=128), XN (n=116), QC (n=102), and NY (n=76) cattle. In the analysis, we used more than 100 heads of cattle for analysis in order to provide more compelling evidence and obtain some statistically significant results. In the JN breed, cattle with a copy number loss type had significantly better traits (P<0.05 or P<0.01) than those with normal and gain types, including body height and body weight (Table 2). Similarly, in the QC breed, cattle with a copy number loss type had a significant body height and hucklebone width than those with normal and gain types (P<0.05 or P<0.01) (Table 2). Among XN individuals, no significant association was found at the *IGF1R* DNA CNV locus with growth traits of 2-year-old cattle (P>0.05) (Table 2), but there was a tendency for loss copy number cases to perform better phenotypic traits than gain or normal copy number cases.

**Table 2:** Association analysis of *IGF1R* DNA copy number variation (CNV) types with growth traits in cattle.

Breeds	Growth traits	CNV type (Mean ± SE)			P value
		Loss	Normal	Gain	
JN	Body height	131.97 ± 1.10${}^{A}$	129.18 ± 0.76${}^{B}$	126.85 ± 0.90${}^{B}$	0.002
	Hip height	134.26 ± 1.33	132.21 ± 0.90	130.25 ± 1.11	0.060
	Body slanting length	154.45 ± 2.12	151.58 ± 1.32	148.25 ± 1.73	0.055
	Chest width	188.55 ± 2.00	186.05 ± 1.81	182.00 ± 1.75	0.077
	Rump length	48.71 ± 0.63	47.86 ± 0.56	47.13 ± 4.16	0.264
	Body weight	420.97 ± 14.16${}^{a}$	399.95 ± 9.24${}^{\mathrm{ab}}$	374.49 ± 11.42${}^{b}$	0.029
QC	Body height	134.91 ± 1.38${}^{A}$	128.86 ± 0.74${}^{B}$	129.05 ± 0.86${}^{B}$	0.003
	Hip height	130.73 ± 1.27	126.27 ± 0.78	126.61 ± 0.91	0.054
	Body slanting length	139.09 ± 2.60	137.29 ± 1.07	138.02 ± 1.17	0.753
	Chest width	179.95 ± 4.05	177.35 ± 1.46	175.57 ± 1.64	0.448
	Rump length	45.27 ± 3.35	44.16 ± 2.63	43.31 ± 2.88	0.092
	Hucklebone width	23.82 ± 1.05${}^{a}$	23.68 ± 0.61${}^{a}$	21.25 ± 0.58${}^{b}$	0.011
	Body weight	421.55 ± 25.43	402.53 ± 8.84	396.77 ± 9.92	0.247
XN	Body height	138.14 ± 1.90	134.61 ± 0.75	134.60 ± 0.45	0.091
	Hip height	140.29 ± 1.74	137.44 ± 0.53	137.77 ± 0.35	0.095
	Body slanting length	161.86 ± 2.44	159.53 ± 1.25	158.38 ± 0.86	0.421
	Chest width	194.43 ± 3.62	192.19 ± 1.43	192.53 ± 1.27	0.868
	Cannon circumference	19.86 ± 0.59	19.22 ± 0.22	18.90 ± 0.15	0.130
	Body weight	572.86 ± 22.69	548.36 ± 10.14	541.14 ± 6.12	0.329

Note: a and b denote values that differ significantly at P<0.05; A and B denote values that differ significantly at P<0.01. JN: loss (31), normal (57), and gain (40). QC: loss (11), normal (49), and gain (42). XN: loss (8), normal (35), and gain (73).

### 3.5 Bioinformatics prediction of *IGF1R* CNV

**Figure 4:**
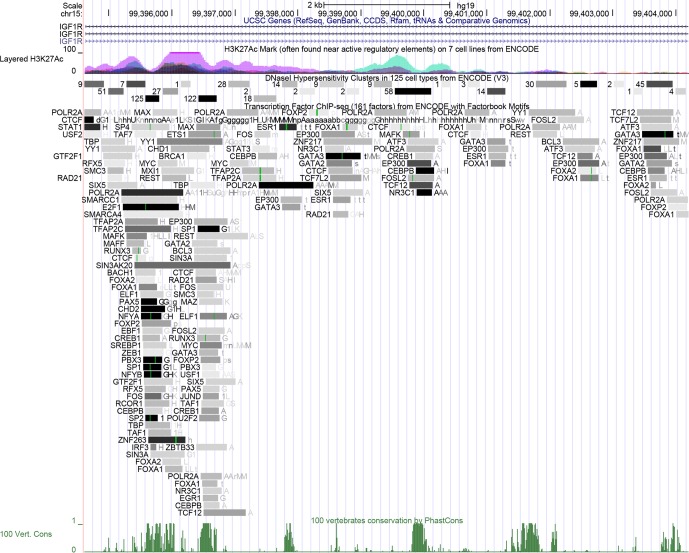
Bioinformatics analysis of *IGF1R* CNV. The peak means histone acetylation above this figure. The rectangle with a number or a letter means DNase I hypersensitive sites or sequence-specific TFs, respectively, and the darker the color, the more likely the combination is. The other peak means similarity between the sequence of *IGF1R* CNV in cattle and the whole-genome sequence in humans at the bottom of this figure.

We found that the similarity between the *IGF1R* CNV sequence in cattle and the whole-genome sequence in humans is 87.5 %, so this region may perform a similar function in the human genome. The result of histone acetylation showed that this region had highly horizontal acetylation. In addition, we found this region had many DNase I hypersensitive sites and transcription factors, and the darker the color, the stronger the ability of transcription factors to bind (Fig. 4).

## 4 Discussion

With the development of genomics, copy number variation has recently been shown to be associated with disease and phenotypic variation (Bujakowska et al., 2017; Keel et al., 2017; Shi et al., 2016). And it had been continuously confirmed to be able to influence individual phenotypic traits through gene dosage or structural variation (Bickhart et al., 2012; Wright et al., 2009). In cattle, previous research has shown that several CNVs were associated with detoxification, immunizing, and signal recognition; these gene receptors include cytochrome P450, ATP-binding cassette (ABC) transporters, and β-defensins (Hou et al., 2011). In addition, the genes *MAPK10*, *LEPR*, and *MICAL-L2* within CNVs could influence Chinese cattle's phenotypic variation (Liu et al., 2016; Shi et al., 2015; Xu et al., 2013).Further experiments were conducted to explore the potential role of candidate gene CNV through a large sample of different cattle breeds.

IGF1 is a protein associated with insulin structure and has an important regulatory role in cell growth, differentiation, and maintaining differentiation, whether before or after birth. It is also an important factor affecting weight (Van Laere et al., 2003). Studies have shown that IGF2 plays an important role in the growth and meat quality of pigs (Maagdenberg et al., 2008). IGF1R is a transmembrane glycoprotein with tyrosine kinase activity in both the IGF1 ligands and the IGF2 ligands. Both need to combine IGF1R play antiapoptotic activity, promoting the transformation of cell mitosis, etc. (Pollak, 2008). So, it can be safely assumed that the *IGF1R* gene may influence bovine phenotype traits. Actually, our results showed that the CNVs of the *IGF1R* gene were significantly associated with body height, body weight, and hucklebone width in Chinese cattle.

Due to the different genetic backgrounds of the four breeds, a large number of specific CNV loci have been detected in cattle (Liu et al., 2010). Among them, compared with the other three varieties, the XN bovine gain type was more than the other variant types, which may be caused by the difference in breeding background between varieties. XN cattle is a type of beef cattle which is a hybrid of French Charolais and Chinese NY cattle, while the other three varieties of yellow cattle belong to Chinese indigenous local yellow cattle. At the same time, the *IGF1R* copy number was dispersed in JN cattle, QC cattle, and NY cattle, indicating that the CNV locus had a large degree of variation among these three cattle breeds, which could further influence the phenotype of cattle.

IGF1R and insulin receptor (IR) is homologous proteins with multiple domains of tyrosine kinase. Both receptors are glycoproteins, which are composed of two α and two β subunits. α-Subunits are extracellular and participate in the ligand binding, while β-subunits contain the transmembrane and intracellular domain (Keyhanfar et al., 2007; Lawrence et al., 2007). IGF1R binds IGF1 with high affinity, while it has a low affinity for IGF2 and insulin (INS). Activated IGF1R is involved in cell growth and survival control. IGF1R is essential for tumor transformation and the survival of malignant cells. Ligand binding activated receptor kinase, which leads to receptor phosphorylation and a variety of substrates of tyrosine phosphorylation as signal transduction proteins including insulin receptor substrate (IRS1/2), Shc, and 14-3-3 proteins. Phosphorylation of IRS protein leads to the activation of two major signaling pathways: the PI3K-AKT/PKB pathway and the Ras-MAPK pathway. The result of activating the MAPK pathway is increased cell proliferation, while activating the PI3K pathway inhibits cell apoptosis and stimulates protein synthesis (Gkioka et al., 2015). So far, there has been little information on IGF1/IGF1R signaling, especially in cattle. In this study, the cattle *IGF1R* gene also showed a broad spectrum in expression, indicating that IGF1R is involved in the transmission and regulation of the growth axis in multiple tissue types. So, it is likely to be involved in a variety of regulation, leading to a change in adult individual metabolic activity and fat deposition ultimately reflected on the phenotype of the cattle. In addition, we think the CNV locus can influence Chinese cattle although it is located in intron 1. With the development of studies, it has been recognized that through the alternative splicing of introns, a single gene can encode many different proteins at the same time. Recently, it has become increasingly clear that introns and their shear processes can influence gene expression through gene transcription, mRNA transport, localization, and translation (Bicknell et al., 2012; Hir et al., 2003).

Dosage effect is one important mechanism underlying the phenotypic effects of CNV. A corresponding missing or amplification may cause function disorder, which lacks a corresponding cause to reduce the level of gene expression. Amplification results in an increase in expression level. Previous studies found that the oncogenes Myc and MYCN were at 17q21.31, chr8q24.22 ∼ 24.23, and significant DNA fragment amplification was observed in this region. Deficiency was observed at 9p21.1 ∼ 9p21.3 and 6p23.1 (Lu et al., 2009). In addition, the dose effect of CNV can also affect the penetrance of genes (Beckmann et al., 2007). Although the correlation analysis was carried out, there is no significant correlation between *IGF1R* CNVs and mRNA expression levels. CNV may influence bovine growth traits by other regulatory mechanisms. Previous studies indicate that CNV may be a key factor in reducing the penetrance of some pathogenic genes and can also significantly influence the expression products of genes by changing their structure. A study found that CNV was associated with the susceptibility of neuroblastoma at lq21.1, which led to changes in the expression of NBPF transcription NBPF23 (Diskin et al., 2009). Meanwhile, bioinformatics predicted that there are a lot of transcription factors in the region, such as FOSL2, SP1, MYC, and E2F1. They play a key role in growth and development (Schwarz et al., 1995; Black et al., 1999; Fan et al., 2014). In addition, there are a lot of DNase I hypersensitive sites and high levels of acetylation in this region, which presumably indicates the presence of enhancer regulation in this region. Moreover, it was found that CNV was not significantly correlated with gene expression, indicating that the copy number variation achieved its phenotypic effects through genes other than *IGF1R*. Further study will be undertaken to outline the underpinning mechanism.

## 5 Conclusions

In conclusion, we determined the *IGF1R* CNV in Chinese cattle breeds. Our study provided a preliminary result for the functional role of the *IGF1R* CNVs in larger populations and different cattle breeds and for a novel and important marker in cattle breeding programs.

## Data Availability

The original data are available upon request to the corresponding author.
